# Ozonation of Amoxicillin and Ciprofloxacin in Model Hospital Wastewater to Increase Biotreatability

**DOI:** 10.3390/antibiotics10111407

**Published:** 2021-11-17

**Authors:** Severina Aleksić, Andreja Žgajnar Gotvajn, Katarina Premzl, Mitja Kolar, Sonja Šostar Turk

**Affiliations:** 1 Faculty of Health Sciences, University of Maribor, Žitna ulica 15, SI-2000 Maribor, Slovenia; severina.aleksic@leone.si; 2 Faculty of Chemistry and Chemical Technology, University of Ljubljana, Večna pot 113, SI-1000 Ljubljana, Slovenia; andreja.zgajnar@fkkt.uni-lj.si; 3 National Laboratory of Health, Environmental and Food, Prvomajska ulica 1, SI-2000 Maribor, Slovenia; katarina.premzl@nlzoh.si

**Keywords:** amoxicillin (AMX), antibiotic, ciprofloxacin (CIP), hospital wastewater, hydrogen peroxide, ozone, sludge, treatment

## Abstract

Amoxicillin (AMX) and Ciprofloxacin (CIP) are antibiotics commonly used in human medicine with high environmental toxicity and poor biodegradability. They have been found in various hospital effluents and groundwater, and their environmental impact is still not fully understood. In this work, we investigated the possibility of treating model wastewaters containing the antibiotics AMX and CIP using ozonation, with the addition of H_2_O_2_ under various conditions, including different pH values, H_2_O_2_, and ozone dosages. The quantification of and treatment efficacy for antibiotic removal were determined via solid phase extraction followed by chromatographic separation by liquid chromatography coupled with tandem triple quadrupole mass spectrometry (LC/MS/MS). This analytical system is quite efficient for the detection of all major antibiotic classes, even if they are present at very low concentrations. The efficiency of ozonation was determined by measuring the TOC (Total Organic Carbon) changes after ozonation of the model wastewater and by measuring the concentration of the two antibiotics. In a sequential activated sludge process of ozone-treated model wastewater, almost complete TOC removal and an overwhelming decrease in antibiotic concentrations (up to 99%) were observed. Ozonation resulted in complete removal of AMX and CIP in less than 30 and 120 min, respectively. The results of this work indicate that ozonation could be a suitable pretreatment method to reduce the toxicity of contaminants (AMX and CIP) and improve the biodegradability of hospital wastewater.

## 1. Introduction

Ciprofloxacin (CIP) from the fluoroquinolone family and Amoxicillin (AMX) from the beta-lactams group are used as broad-spectrum antimicrobial agents in hospitals, households, and veterinary medicine to treat bacterial infections. These applications result in the significant contamination of wastewater and groundwater. Both substances can enter the environment through a variety of pathways, including human excreta, improper disposal of unused medications, industrial and hospital wastewater, and veterinary use. One of the most important sources of these substances is wastewater [1,2].

AMX and CIP are highly toxic and difficult to biodegrade. The treatment of wastewater containing antibiotics is very complex because these wastewaters contain not only antibiotics but also inorganic and organic compounds, which all together can inhibit the activity of microorganisms in wastewater treatment plants (WWTP). The inhibitory effect on microorganisms in WWTPs is mainly seen in conventional WWTPs that use the activated sludge process, biological filters, or membranes to treat wastewater. Conventional wastewater treatment plants are not primarily designed to treat biologically active substances; these are inadequately removed from the wastewater and, thus, enter the aquatic environment [1,2].

Therefore, the removal of CIP in different types of biological treatment plants, e.g., activated sludge and rotating biological contactors, can reach up to 59% and 76%, respectively. However, the final concentrations in treated wastewater are still problematic (µg L^−1^), as both CIP and AMX occur in the aquatic environment at concentrations up to 400 µg L^−1^ [3]. Although measured concentrations are generally many times lower than therapeutic doses and are not acutely toxic, little is known about the long-term effects on aquatic organisms. Moreover, previous reports have indicated that CIP and AMX may specifically trigger microbial communities in aquatic ecosystems, contributing to the development of resistant bacteria [4,5,6]. Therefore, much research effort has been made in the last decade to find efficient methods to remove these substances from wastewater. There are several methods that give promising results and belong to a group of AOPs (Advanced Oxidation Processes) [7,8].

AOPs rely on the formation of hydroxyl radicals (•OH), which can oxidize organic molecules in a strongly nonselective manner [5]. Recent studies show that AOPs can be successfully used for the treatment of pharmaceutical wastewater. Oxidation by Fenton and photo-Fenton processes could degrade Sulfamethoxazole very effectively [9]. The fate of pharmaceutical Diclofenac removal by photolysis and H_2_O_2_ enhanced photolysis has been reported [10,11]. The combined UV/H_2_O_2_ process was also very effective in degrading Diclofenac [12]. Reverse osmosis, activated carbon, and ozonation have been shown to significantly reduce or eliminate antibiotics and pharmaceutical substances in wastewater. These processes use one or more oxidants, usually hydrogen peroxide and/or oxygen [12,13]. AOPs have the advantage of generally eliminating such contaminants through mineralization or conversion to products that are less harmful to human health and the aquatic environment [14].

Ozonation is one of the most efficient AOPs. Ozone and/or hydroxyl radicals passivate the bactericidal properties of antibiotics by impairing or modulating their pharmaceutically active functional groups, such as the N-etheroxime and dimethylamino groups of Macrolides [15]. High removal rates (90%) were achieved by ozonating the compounds with electron-rich aromatic systems such as hydroxyl, amino (e.g., sulfamethoxazole), acylamino, alkoxy, and alkyl aromatic groups, as well as the compounds with deprotonated amine groups (e.g., erythromycin, ofloxacin, and trimethoprim) and non-aromatic alkene groups, since these structural components are highly modifiable for oxidative attack [16]. The ozonation process was found to be effective for the removal of beta-lactams, macrolides, sulfonamides, trimethoprim, quinolones, tetracyclines, and lincosamides [17].

However, due to the high concentrations and diversity of organic compounds in wastewater, mineralization of the active compounds is often incomplete [18], and such oxidized byproducts can lead to a significant increase in toxicity compared to the original compound [19]. Moreover, many studies only focus on degradation kinetics and degradation processes without determining toxicity. Very little is currently known about the transformation products, the specific reaction mechanisms, and the toxicity assessment of the transformation mixtures. These assessments are of considerable importance for environmental protection and wastewater treatment. Therefore, the primary objective of this study was to use an AOP (i.e., ozonation) with the addition of hydrogen peroxide to remove and inactivate high concentrations of CIP and AMX in model wastewaters. Such highly contaminated wastewaters are usually generated in small quantities, so ozonation seems to be a viable option for their treatment. The main advantage of combining ozone with hydrogen peroxide is the accelerated formation of •OH and, thus, better oxidation of the active substances [20].

Since ozonation does not necessarily lead to complete mineralization of the antibiotic components, we also determined the concentrations of CIP and AMX as well as total organic carbon (TOC) before, during, and after ozonation to assess whether the components were completely degraded [21]. In addition, the toxicity to the activated sludge was monitored before and after the experiment to evaluate the possible production of toxic byproducts that could further affect the biological treatment plant.

A recent review found that this area is still of great interest because conventional wastewater treatment plants cannot effectively remove antibiotics. Moreover, the occurrence of antibiotics in wastewater is of concern worldwide. The most promising methods for treating antibiotics are ozone-based AOPs, as they are fast, non-selective, and effective [22,23,24,25,26].

In our previous work [27], we studied the removal of AMX and CIP from hospital wastewater by subcritical and supercritical water oxidation. We observed the effect of temperature and flow rate of the sample on the concentration of antibiotics. We concluded that the highest chemical oxygen demand (COD) and TOC removal was achieved at the highest temperature of 500 °C, where it was reduced by 76% and 63%, respectively. An additional toxicity test, measuring the respiration inhibition of the activated sludge, confirmed that the samples after subcritical and supercritical oxidation were less inhibitory than the initial solution. Despite the encouraging results, we concluded that this process is not suitable for industrial or commercial use due to the high investment and operating costs.

## 2. Results

### 2.1. LC/MS Methodology/Optimization

Chromatography of the selected antibiotics was tested on three different columns: ASCENTIS Express C18 (50 mm × 2.1 mm I.D., 2.7 µm), Synergie Fusion-RP 100 A (50 mm × 2.0 mm I.D., 2.5 µm), and Kinetex XB-C18 100 A (50 mm × 2.1 mm I.D., 2.6 µm). The shapes of the chromatographic peaks were comparable, although the peak intensity was about 20% higher on the Synergi Fusion-RP 100 column. The analysis of the compounds was therefore performed using a Synergie 2.5 µm Fusion RP 100 A, 50 × 2.0 (Phenomenex, Germany), a polar end-capped C18 phase column (it contains high reproducibility and a stable phenyl phase), which improves the retention of highly polar and aromatic compounds. For optimal chromatographic separation and good peak shape, 0.1% formic acid was added to promote protonation of both compounds and increase sensitivity. The retention time of amoxicillin was 1.12 min, while the retention time of ciprofloxacin was 7.08 min. LOD (limit of detection) and LOQ (limit of quantification) values for ciprofloxacin were found to be 0.07 µg L^−1^ and 0.23 µg L^−1^, respectively. LOD and LOQ values for amoxicillin were found to be 0.05 µg L^−1^ and 0.15 µg L^−1^, respectively.

The linearity of the method was checked daily by constructing a calibration curve at six concentration levels. Calibration curves were constructed from the measured areas of the chromatographic peaks by plotting the concentration ratio of the standards to the internal standard on the *x*-axis and the corresponding area ratio on the *y*-axis.

**Figure 1:**
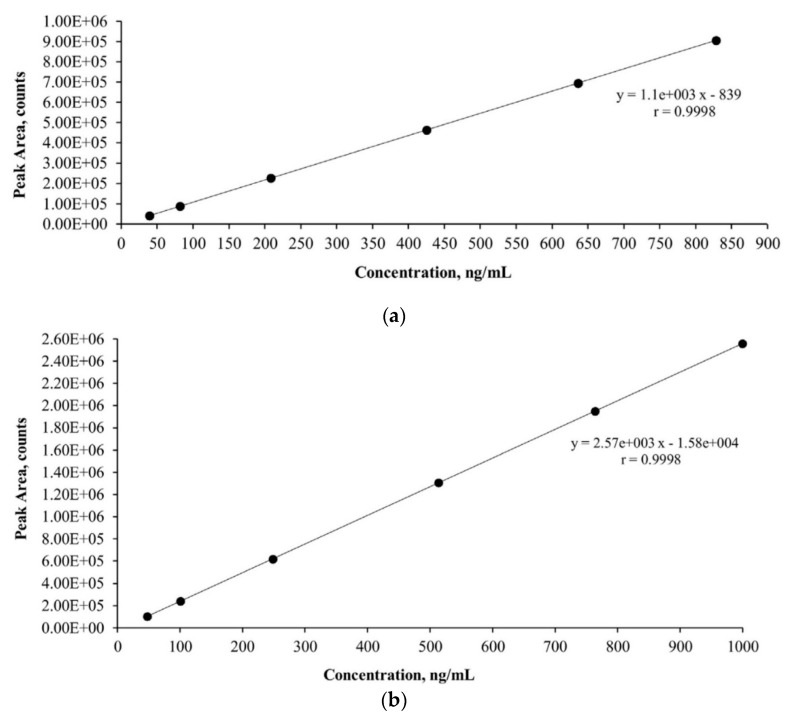
(**a**) Linear response of AMX. (**b**) Linear response of CIP.

The linear calibration curves obtained for AMX and CIP are shown in Figure 1.

Figure 1 shows the calibration curve of AMX, ranging from 40 to 800 ng mL^−1^, and the calibration curve of CIP, ranging from 50 to 1000 ng mL^−1^. The calibration curves obtained between the average peak and concentration showed a linear relationship with a correlation coefficient of 0.9998 for both antibiotics, so the LC/MS method was found to be highly selective and reproducible.

### 2.2. Amoxicillin and Ciprofloxacin Degradation

**Figure 2:**
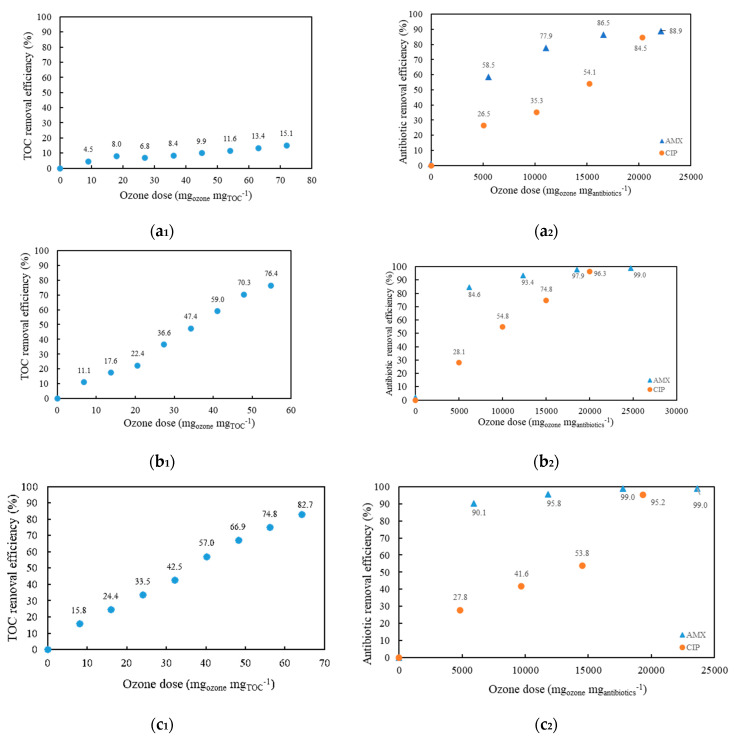
TOC concentration of model wastewater (**a_1_**) and AMX/CIP concentration at pH = 3.70 (**a_2_**). TOC concentration of model wastewater (**b_1_**) and AMX/CIP concentration at pH = 10.85 (**b_2_**). TOC concentration of model wastewater (**c_1_**) and AMX/CIP concentration at pH = 10.85 with addition of H_2_O_2_ (**c_2_**).

The effectiveness of AMX and CIP ozonation was evaluated considering the influence of two variables: pH and the addition of hydrogen peroxide. Figure 2a_1_,b_1_,c_1_ shows the TOC removal efficiency for model wastewater with AMX and CIP. It can be seen that alkaline conditions (Figure 2b_1_) showed a higher removal efficiency compared to acidic conditions (Figure 2a_1_). The TOC removal efficiency was five times more efficient. As shown in Figure 2a_2_,b_2_,c_2_, ozonation was effective in removing AMX and CIP from the model wastewater. For both CIP and AMX, degradation was most pronounced under alkaline conditions (pH = 10.85), with the combined O_3_/H_2_O_2_ process achieving 99% removal efficiency for AMX and 95% for CIP. Regardless of the process conditions, the total concentration of CIP (Figure 2a_2_,b_2_) remained constant in all cases during the first 30 min (5000 mg_ozone_ mg_antibiotics_^−1^), and then removal efficiency increased to 35.3–54.8% at an ozonation time of 60 min (10,000 mg_ozone_ mg_antibiotics_^−1^), while the concentration of AMX increased to 78% at 60 min (11,059 mg_ozone_ mg_antibiotics_^−1^) in acidic conditions and to 85% at 30 min (6179 mg_ozone_ mg_antibiotics_^−1^) in alkaline conditions.

The addition of H_2_O_2_ also slightly accelerated CIP and AMX degradation (Figure 2c_2_). The efficiency of degradation of the compounds gradually increased with increasing H_2_O_2_ concentration. Figure 2a_2_ also shows that at pH 3.70, the degradation of both AMX and CIP takes 120 min (22119 mg_ozone_ mg_antibiotics_^−1^ and 20338 mg_ozone_ mg_antibiotics_^−1^, respectively) after receiving the same ozone dose. The difference occurs at a pH of 10.85, where CIP degradation takes twice as long as AMX degradation.

It can be concluded that both acidic and alkaline conditions (pH 3.70 and pH 10.85) increased the degradation efficiency. However, the concentrations of both antibiotics remained higher at pH 3.70 than at pH 10.85, and the removal of TOC was also more effective at pH 10.85 than at pH 3.70.

### 2.3. TOC Degradation

**Figure 3:**
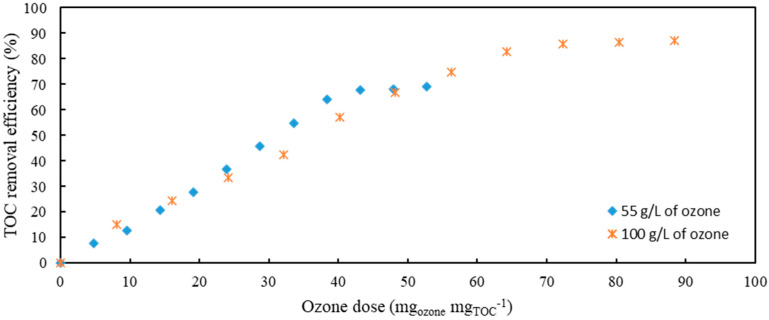
Comparison of different concentrations of ozone for ozonation of model wastewater containing AMX and CIP.

Comparison of two different applied ozone gas flows resulting in two concentrations of ozone in gaseous phase (Figure 3) showed that the results are comparable. However, it can be seen that at an applied concentration of 100 g L^−1^ of ozone, 90% TOC was removed after 120 min (72 mg_ozone_mg_TOC_^−1^), while at 55 g L^−1^, the removal efficiency reached only 70% after 120 min (43 mg_ozone_mg_TOC_^−1^).

**Figure 4:**
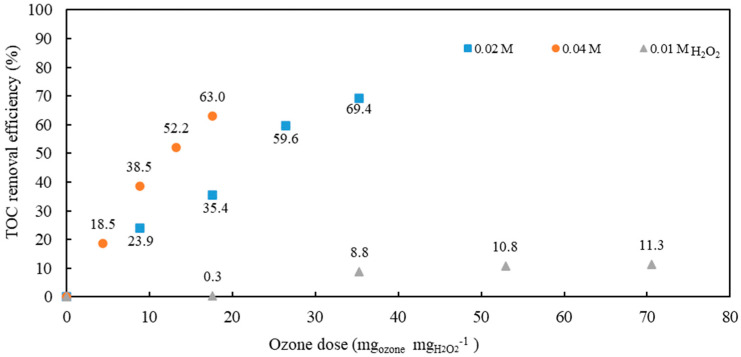
TOC removal efficiency at pH = 10.85 and different dosages of H_2_O_2_ in model wastewater containing AMX and CIP.

In the next set of experiments, ozonation with the addition of H_2_O_2_ (0.01, 0.02, and 0.04 M) was studied (Figure 4).

As observed, TOC removal efficiencies increased with hydrogen peroxide concentrations of 0.02 M and 0.04 M.

### 2.4. Toxicity Test

**Figure 5:**
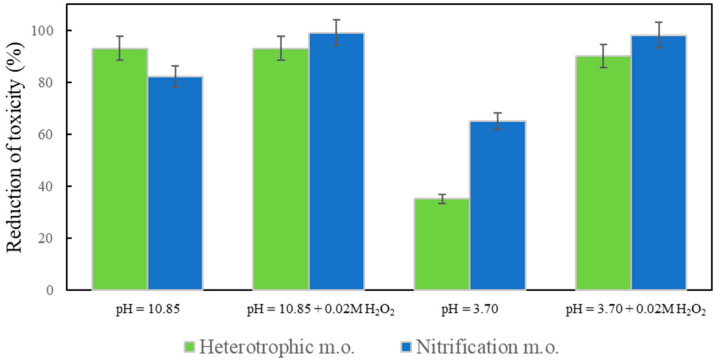
Reduction of toxicity of model wastewater after 120 min of ozonation at pH 3.70 and 10.85 with and without addition of 0.02 M H_2_O_2_.

The toxicity of ozonated samples at pH 3.70 and pH 10.85 with and without the addition of H_2_O_2_ (model wastewater with both antibiotics) was evaluated (Figure 5).

The toxicity was calculated as the relative reduction compared to the untreated model wastewater (%). After ozonation under different conditions, toxicity was always reduced; it had a very low effect on heterotrophic and nitrifying microorganisms of the activated sludge, confirming the deactivation of both antibiotics. To improve nitrification efficiency in biological wastewater treatment plants, it is very important to reduce the inhibition of nitrifying microorganisms, which are sensitive to antibiotics. Ozone and/or hydroxyl radicals passivate the bactericidal properties of antibiotics by interfering or modulating their pharmaceutically active functional groups [28].

## 3. Discussion

The extraction efficiency of the selected antibiotics was tested on three different SPE cartridges, and the results showed (data not shown) that the Oasis HLB cartridge gave the best results at a set pH of 7.00. Similar results have also been reported by other authors [29]. Based on these results and the corroborating literature [30,31], the Oasis HLB cartridge (200 mg/6 mL) was selected for effluent monitoring. Then, LC/MS optimization was performed, where an external solution with diluted antibiotics was used for sample quality control. Detection was performed by multiple reaction monitoring (MRM) with positive electrospray ionization (ESI+).

The effectiveness of AMX and CIP ozonation was then evaluated. The acidic conditions favor a direct reaction of ozone, while the alkaline conditions support the formation of •OH radicals, and thus, the oxidation of organic molecules proceeds much faster compared to systems with ozone alone [32]. Similarly, the addition of H_2_O_2_ prior to ozonation accelerates the formation of •OH radicals, so the removal efficiencies may be comparable [33]. Despite different degradation rates during ozonation of model wastewaters, the studied antibiotics were almost completely degraded (more than 80%). From the TOC measurements, it can be concluded that some byproducts still remained.

Similarly to previous studies [34], high pH values (> 10) cause high efficiency of ozonation and rapid degradation of organic matter in wastewater due to the formation of highly reactive •OH radicals (Figure 2a_2_,b_2_,c_2_) [35]. In their study, Akmehmet and Ötker [36] applied ozonation to synthetic Penicillin wastewater. About 70% and 40% of the initial COD (450 mg L^−1^) and TOC (162 mg L^−1^) were removed by ozonation after 1 h at an applied ozone dose of 2.96 g L^−1^ at pH values of 7.00 and 11.00 and a temperature of 20 °C, respectively. As shown in our case, the removal of TOC (Figure 2a_1_,b_1_,c_1_) was more efficient at higher pH values.

The role of hydrogen peroxide has been highlighted in several previous works—a high concentration of hydrogen peroxide accelerates the ozonation reaction and provides high contaminant removal efficiency. This was probably caused by both the self-decomposition of H_2_O_2_ into oxygen and water and the recombination of ∙OH to ∙O_2_H, resulting in a higher oxidation efficiency [34,35].

The mechanisms of AMX degradation during ozonation have previously been described by Andreozzi et al. [10]. It was found that the reaction rate between AMX and molecular ozone is strongly pH dependent, from 4 × 10^3^ M^−1^ s^−1^ at pH 2.50 to 6 × 10^6^ M^−1^ s^−1^ at pH 7.00. The ozone attack is mainly directed towards the phenolic ring, leading to the formation of hydroxyderivative intermediates. This was also confirmed in our study, as TOC was reduced less (69%) than the concentration of antibiotics (90%).

The mechanisms of CIP degradation during ozonation have previously been described by Demeestere et al. [37]. At pH 10.00, deprotonation of the N4’atom of the piperazinyl group enhanced direct ozonation at this site of the molecule. The addition of H_2_O_2_ to the CIP ozonation experiments at pH 7.00 had limited effect on quinolone degradation and ozone and H_2_O_2_ consumption, suggesting that the radical chain mechanism is of lesser importance for quinolone degradation compared to direct ozonation. Identification of the degradation products showed the strongest degradation at the piperazinyl substituent at pH 10.00, while degradation at the quinolone moiety at pH 7.00 appears promising.

The mechanisms of these reactions have not been fully elucidated, and there is considerable disagreement in the literature as to the exact intermediates that are formed, including whether or not the hydroxyl radical is an intermediate [37].

Demeestere et al. [37] additionally performed a toxicity assay after the ozonation of CIP. The residual antibacterial activity against *P. fluorescens* and *E. Coli* appeared to be mainly determined by the rate of degradation of the parent compound. For *B.coagulans*, there was no difference in the reduction of antibacterial activity, although the fastest ozonation was achieved at pH 10.85.

The literature on the removal of antibiotics by ozonation from heavily polluted wastewater is currently limited. Most ozonation experiments have been conducted under controlled conditions and with antibiotics dissolved in deionized water. For example, Najafpoor et al. [38] determined the efficiency of CIP removal using ozonation from aqueous solutions. The process parameters were studied with CIP concentrations of 10–50 mg L^−1^, pH of 3–12, reaction time of 60 min, and ozone concentration of 1.4 mg L^−1^ min^−1^ in a semiconductor reactor. The results showed that under optimal conditions (pH = 12 O_3_ = 1.4 g L^−1^ min^−1^ and an initial antibiotic concentration of 10 mg L^−1^), 94.6% of CIP was removed. In our study, 1 mg L^−1^ CIP was efficiently removed from the model wastewater at a higher pH (10.85).

Lefebvre et al. [39] studied the suitability of ozone pretreatment for AMX wastewater before biological treatment. They found that ozonation is not a suitable pretreatment for AMX containing pharmaceutical wastewater. De Witte et al. [40] studied the effect of pH on CIP degradation during ozonation of hospital wastewater. Degradation at pH 7.00 increased the half-life of CIP to 29.1 min, compared to 26.8 min at pH 3.00 and 18.7 min at pH 10.00.

Our results are in agreement with those of Zaviska et al. [41], who also showed that the combination of H_2_O_2_ and O_3_ produced a higher number of very reactive •OH radicals and made the process more efficient. Due to the high cost of ozone generation, this combination also makes the process economically feasible [42].

## 4. Materials and Methods

### 4.1. Chemicals

Ciprofloxacin (analytical standard, 99.5% purity, C_17_H_18_FN_3_O_3_, 331.34 g mol^−1^), Amoxicillin trihydrate (analytical standard, 85.7% purity, C_16_H_19_N_3_O_5_S·3H_2_O, 419.45 g mol^−1^), and Ciprofloxacin-D8 hydrochloride hydrate (99% purity) were provided by Sigma-Aldrich, Fluka (Darmstadt, Germany). Amoxicillin (3H_2_O Phenyl-13C6, ≥95% purity) was obtained from USA Cambridge Isotope Laboratories (Andover MA, USA). All reagents were purchased in HPLC grade from Sigma-Aldrich, Fluka (Darmstadt, Germany) or Merck (Darmstadt, Germany). Hydrogen peroxide (30% *w/w*, Ph.Eur., USP, pharmaceutical grade) was purchased from AppliChem, Darmstadt, Germany.

### 4.2. Model Wastewater Preparation

Synthetic standard wastewater (ISO OECD standard, 2004) was prepared from Solution 1 (containing urea (30 g L^−1^), sodium chloride (NaCl, 7 g L^−1^), magnesium sulfate heptahydrate (MgSO_4_·7H_2_O, 2 g L^−1^), and potassium dihydrogen phosphate (KH_2_PO_4_, 28 g L^−1^)) and Solution 2 (containing calcium chloride monohydrate (CaCl_2_·H_2_O, 4 g L^−1^)). Then, 1 mL of Solution 1 and 1 mL of Solution 2 were mixed with 160 mg of peptone in 1 L of deionized water. This mixture gives an average TOC concentration of about 78–82 mg L^−1^ and COD concentration of 356–386 mg L^−1^. An appropriate volume of the mixture of the standard solution of Ciprofloxacin and Amoxicillin was added to the standard synthetic municipal wastewater to obtain a concentration of 1 mg L^−1^ of AMX and 1.2 mg L^−1^ of CIP.

### 4.3. Ozonation Experiments

**Figure 6:**
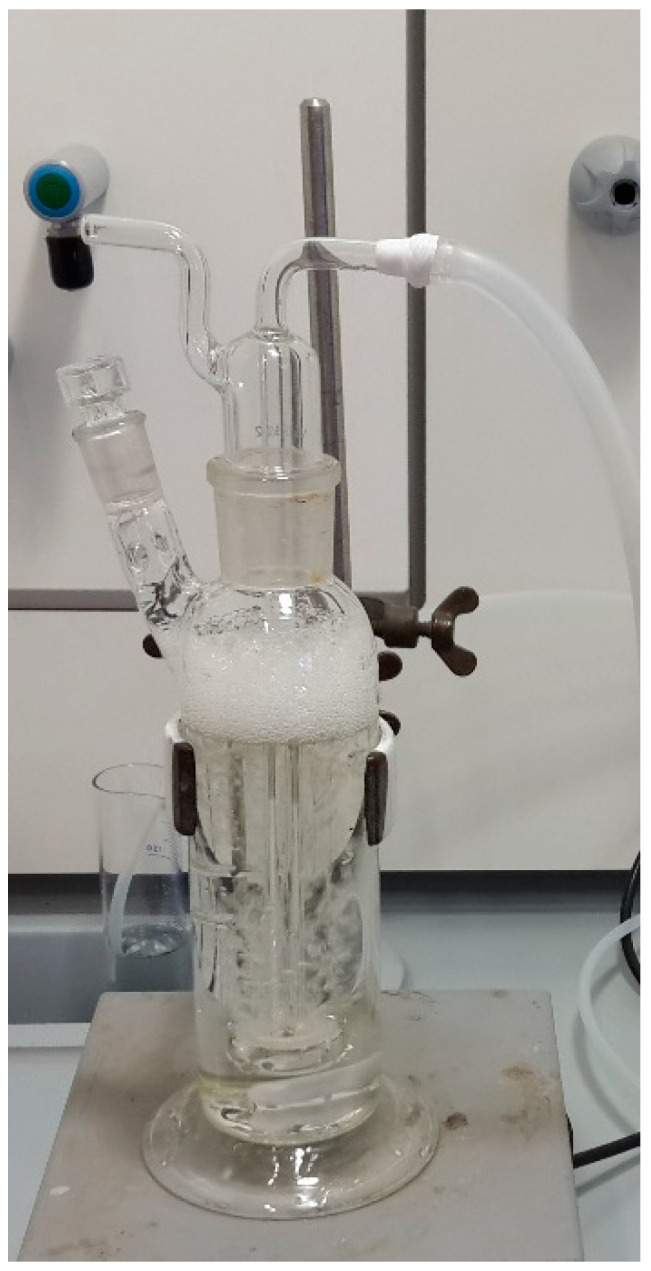
Glass bubble column reactor.

The main experiment was set up to study the effect of ozone dosage on treatment performance. Ozone was generated from pure oxygen used as feed gas (>99.5 vol.%, Messer, Bad Soden, Germany) and was introduced at the bottom of the glass bubble column reactor (250 mL) (Figure 6) through a gas distributor using the Wedeco ozone generator (Xylem Water Solutions Herford GmbH, type OCS Modular 8 HC, Herford, Germany). The O_3_ generator used had a production capacity of 8 g h^−1^, with the possibility to control the gas flow at Q_G_ = 10–100 NL h^−1^ and to adjust the ozone concentration in the produced gas to 10–100 g Nm^−3^. All gas phase flows (Nm^−3^, NL) were measured with scales of normal temperature (0 °C, 273 K) and pressure (100 kPa) conditions.

All ozonation experiments were conducted for 120 min. At *t* = 0, 15, 30, 45, 60, 75, 90, 105, and 120 min, 5 mL of the sample was taken for determination of TOC, and at *t* = 30, 60, 90, and 120 min, the samples were analyzed for the concentrations of AMX and CIP. The experiments were performed in duplicate, and analyses were repeated three times. The experimental conditions with ozone doses are given in Table 1 below. Ozonation was performed under acidic conditions (pH = 3.70, Table 1, Value ^1^) and alkaline conditions (pH = 10.85) with (Table 1, Value ^3^) and without (Table 1, Value ^2^) the addition of hydrogen peroxide.

To obtain the most efficient treatment performance, we tried different doses of H_2_O_2_ (Table 1, Value ^5^) and different O_3_ doses (Table 1, Value ^3^).

The working pressure was 0.5 bar and the gas flow was 30 L h^−1^. The experiments were carried out at room temperature (20 ± 1 °C). Prior to ozonation, the pH of the process water was adjusted to either pH 3.70 ± 0.10 or pH 10.85 ± 0.10 by adding sulfuric acid (H_2_SO_4_) or sodium hydroxide (NaOH). Hydrogen peroxide (30%, Ph.Eur., USP, pharma grade, Applichem, Darmstadt, Germany) was dosed into the system at the beginning of the experiment, to combine O_3_ with H_2_O_2_.

### 4.4. Analytical Procedure

#### 4.4.1. High-Performance Liquid Chromatography Combined with Mass Spectrometry (HPLC/MS)

We wanted to develop a method that would identify CIP and AMX simultaneously. The content of both in the wastewater was determined by liquid chromatography combined with tandem mass spectrometry (LC/MS/MS). The method proved to be highly selective and highly sensitive. To develop the method, antibiotic standard solutions were prepared and injected directly into the mass spectrometer (AB Sciex API 2000, SCIEX, Framingham, MA, USA). We also optimized the conditions at the mass detector (ionization, capillary tension, source temperature, and collision energy). During optimization, we tended to obtain the most intense reaction for both base and fragment ionization.

For the optimization of the MS detector, we optimized the following parameters (as shown in Table 2): DP (Declustering Potential), CE (Collision Energy), and CXP (Collision Cell Exit Potential).

The selected antibiotics were then analyzed using a 50 mm × 2.0 mm Synergy FusionRP column with 2.5 µm particle size (Phenomenex, Aschaffenburg, Germany). The following mobile phase gradient was used with Solvent A (95/5/0.2, *v/v/v*; a mixture of ultrapure water, acetonitrile, and formic acid) and Solvent B (50/50/0.2, *v/v/v*; a mixture of methanol, acetonitrile, and formic acid). The gradient was as follows: 0 min, 0% B; 2 min, 0% B; 7 min, 100% B; 7.10, min 0% B; and 17 min, 0% B. The solvents were pumped through the column at a flow rate of 300 µL min^−1^. The injection volume was 50 µL and the column temperature was maintained at 40 °C. The LC/MS/MS method and all optimizations were performed using a pure solution of AMX and CIP at a concentration of 200 ng mL^−1^ in a solvent of 0.1% formic acid in a mixture of CH_3_OH:CH_3_CN (1:1). Chromatograms were also recorded at a flow rate of 300 µL min^−1^ using two other columns: ASCENTIS Express C18 (50 mm × 2.1 mm I.D., 2.7 µm) and Kinetex XB-C18 100 A (50 mm × 2.1 mm I.D., 2.6 µm).

The model wastewater samples were prepared in replicates. AMX and CIP were spiked at 850 ng mL^−1^ because the quantification limits in the municipal and heavily contaminated hospital wastewater samples were higher and the linearity ranges were different. For quality control, an external 6-point calibration curve in the range 40 ng mL^−1^–800 ng mL^−1^ was constructed and measured with the assay samples. The squared coefficient of determination for selected compounds was determined by the quadratic regression of the calibration curves (r^2^ > 0.99).

The extraction efficiency of the selected antibiotics was tested on three different SPE cartridges: Varian Bond Elut Plexa, 60 mg/3 mL; Oasis HLB, 200 mg/6 mL; and Supelco HLB Select, 60 mg/3 mL.

Solid phase extraction was then performed using the Oasis HLB 200 mg column. The procedure was as follows: 200 mL of the wastewater sample was adjusted to pH 8 (with 0.05 M NH_4_OH) and 10 μL ISTD MIX 2 (c = 200 ng mL^−1^) was added.

Conditioning of the selected Oasis HLB 200 mg cartridge was performed by first passing three volumes of MeOH and three volumes of Mili-Q water (which was previously adjusted to pH 8) through the column. The prepared wastewater sample was loaded onto the SPE cartridge at a flow rate of 3 mL min^−1^, then washed with 10 mL of 10% MeOH in water and dried with a gentle stream of air. Finally, elution was performed with two volumes of MeOH. The extracts were collected in 10 mL plastic tubes. The eluate was then concentrated to approximately 300 µL under a gentle air flow. The samples were then made up to 1 mL in a test tube containing a solvent consisting of Mili-Q water and a 95:5 mixture of methanol and acetonitrile. Finally, the samples were centrifuged at 4000 rpm for 10 min.

#### 4.4.2. TOC Determination

To monitor the ozonation efficiency of the selected antibiotics in the model effluent, TOC concentrations (mg L^−1^) were determined according to DIN EN 1484 [43] using Shimadzu TOC 5000A analyzer (Shimadzu, Kyoto, Japan).

### 4.5. Toxicity Assays

In order to assess the residual toxicity of the treated wastewater, the inhibition of oxygen consumption by nitrifying and heterotrophic microorganisms in the activated sludge was measured according to ISO 8192:2007 [28]. Activated sludge consumes oxygen through the degradation of added readily biodegradable substances (peptone). If the wastewater contains toxic substances, the oxygen consumption rate is reduced. The activated sludge for the test was taken from a wastewater treatment plant treating mostly domestic wastewater (350,000 PE). The sludge was washed three times with tap water to remove organic substrate from the wastewater. Then, the activated sludge was aerated and stirred for 24 h. The concentration of the activated sludge was determined by filtering through filter paper and drying at 105 °C to constant mass. In the toxicity test, an appropriate amount (mL) of the sludge was added to achieve a concentration of 1500g_MLVSS_L^−1^. Different mixtures were prepared: (i) 70 vol.% of the model wastewater containing 1.2 mg L^−1^ antibiotics, (ii) 70 vol.% of the ozone treated model wastewater, and (iii) test system without the sample (blank).

Oxygen consumption rates were calculated from the measured oxygen concentrations (mg_oxygen_L^−1^) as a function of time (30 min) using a CellOx 325 oxygen electrode (WTW a xylem brand, Xylem Inc., New York, NY, USA). The oxygen consumption rates of the samples were compared with the blank sample to determine the inhibition of oxygen consumption (%).

The blank sample and the model wastewater samples were continuously aerated on a magnetic stirrer (RH DW Ika, Staufen, Germany) for 30 min. After 30 min, each mixture was transferred to the closed 300 mL oxygen bottle. Over 6 min, the dissolved oxygen concentration was measured at 30 s intervals using the oxygen electrode. The abiotic sample was aerated without the addition of activated sludge. The inhibition test was performed at a temperature of 20 ± 2 °C.

## 5. Conclusions

In the last decade, antibiotics have been found in many aquatic ecosystems, causing various adverse effects on aquatic organisms. To protect the environment and human health, their introduction into the environment should be avoided. One of the ways to do this is to improve biological wastewater treatment by adding an additional stage that would ensure more efficient treatment. The present study has shown that CIP and AMX can be successfully removed by ozonation (at a dosage of 100 mg L^−1^) under alkaline conditions. Removal efficiencies were 99% for AMX and 96% for CIP, but some degradation products remained, as confirmed by TOC analyses. The addition of H_2_O_2_ slightly increased the TOC removal efficiency, although the overall amount of antibiotic removal remained the same as without addition. The results also showed that the toxicity of the activated sludge to heterotrophic and nitrifying microorganisms of the activated sludge was significantly reduced by ozonation, so further biological treatment could be considered. The addition of hydrogen peroxide improved the process. Therefore, ozonation is a promising technique for reducing the toxicity of wastewater containing persistent antibiotics that are difficult to biodegrade and could be used in the pretreatment system before the conventional biological treatment plant.

## Figures and Tables

**Table 1:** Experimental conditions.

Parameter	Value ^1^	Value ^2^	Value ^3^	Value ^4^	Value ^5^
pH (/)	3.70 ± 0.10	10.85 ± 0.10	10.85 ± 0.10	10.85 ± 0.10	10.85 ± 0.10
Applied O_3_ concentration (mg L^−1^)	100	100	55 and 100	100	100
Applied H_2_O_2_ concentration (g L^−1^)	-	-	-	0.68	0.34, 0.68,1.36
Ozone dose (mg_ozone_mg_TOC_^−1^)	0, 18, 36, 54, 72	0, 14, 27, 41, 54	0, 9.6, 19, 28, 8, 38, 53	0, 16, 32, 48, 64	-
Ozone dose (mg_ozone_ mg_antibiotics_^−^^1^)	0, 5000, 10,000, 150,000, 20,000, 25,000	0, 5000, 10,000, 150,000, 20,000, 25,000	0, 16, 32, 48, 64, 88	0, 5000, 10,000, 150,000, 20,000, 25,000	-
Reaction time (min)	120	120	165	120	120
Mixing speed (rpm)	200	200	200	200	200
Sample volume (mL)	250	250	250	250	250

^1^ The experimental conditions for ozonation in acidic conditions. Applied ozone doses ranged from 9 mg_ozone_mg_TOC_^−1^ (*t* = 15 min) to 72 mg_ozone_mg_TOC_^−1^ (*t* = 120 min) and from 5084 mg_ozone_ mg_antibiotics_^−1^ (*t* = 30 min) to 20,339 mg_ozone_ mg_antibiotics_^−1^ (*t* = 120 min). ^2^ The experimental conditions for ozonation in alkaline conditions. Applied ozone doses ranged from 6.8 mg_ozone_ mg_TOC_^−1^ (*t* = 15 min) to 54.8 mg_ozone_ mg_TOC_^−1^ (*t* = 120 min) and from 5000 mg_ozone_ mg_antibiotics_^−1^ (*t* = 30 min) to 25,000 mg_ozone_ mg_antibiotics_^−1^ (*t* = 120 min). ^3^ The experimental conditions for the O_3_ process with the addition of different O_3_ doses. Applied ozone doses ranged from 6.8 mg_ozone_ mg_TOC_^−1^ (*t* = 15 min) to 54.8 mg_ozone_ mg_TOC_^−1^ (*t* = 120 min) and from 5000 mg_ozone_ mg_antibiotics_^−1^ (*t* = 30 min) to 25,000 mg_ozone_ mg_antibiotics_^−1^ (*t* = 120 min). ^4^ The experimental conditions for ozonation in alkaline conditions with the addition of H_2_O_2_. Applied ozone doses ranged from 8.0 mg_ozone_ mg_TOC_^−1^ (*t* = 15 min) to 64.3 mg_ozone_ mg_TOC_^−1^ (*t* = 120 min) and from 5000 mg_ozone_ mg_antibiotics_^−1^ (*t* = 30 min) to 25,000 mg_ozone_ mg_antibiotics_^−1^ (*t* = 120 min). ^5^ The experimental conditions for the O_3_ process with the addition of different H_2_O_2_ doses.

**Table 2:** Optimization parameters DP, CE, CXP for standards.

Compound	Transition	DP (V)	CE (V)	CXP (V)
Ciprofloxacin	332.16/231.2	56	51	4
Ciprofloxacin	332.16/288.2	56	23	4
Amoxicillin	366.171/114.1	31	25	0
Amoxicillin	366.171/208.2	31	19	4

## Data Availability

Not applicable.
